# Analysis of Lymphocyte Immunological Reactivity in Patients with Pleural Effusions of Different Aetiology

**DOI:** 10.3889/oamjms.2016.009

**Published:** 2015-12-25

**Authors:** Zlatica Goseva, Biserka Jovkovska Kaeva, Angelko Gjorcev, Elena Jovanovska Janeva, Zoran Arsovski, Sava Pejkovska, Aleksandra Tatabitovska

**Affiliations:** *University Clinic of Pulmonology and Allergology, Faculty of Medicine, Ss Cyril and Methodius University of Skopje, Skopje, Republic of Macedonia*

**Keywords:** pleural effusions, lymphocyte, CD markers, malignant pleural fluid, tuberculous pleural effusions

## Abstract

**BACKGROUND:**

The proportion of T and B lymphocytes in pleural fluids and blood may point to the presence of local immunological phenomena in pleural disorders.

**AIM:**

Aim of study was to evaluate the lymphocyte phenotype and the ratio between helper (CD4+) and cytotoxic/suppressor (CD8+) lymphocytes in malignant and non-malignant effusions.

**MATERIAL AND METHODS:**

We studied 48 patients with pleural effusions. First group had 18 patients with tuberculosis pleural effusions; second group had 20 patients with malignant pleural fluids, third group had 10 patients with transudates and 30 healthy controls. We investigated the distribution of T and B lymphocytes, T cells with helper/inducer CD4 or suppresser/cytotoxic CD8 phenotypes and the CD16 subset.

**RESULTS:**

Results showed decreases levels of CD3, CD4, and CD16 T cells in blood of patients versus healthy controls. There were increases in the percentage of the CD3 and CD4 T cells in the pleural fluid compared with values in the blood with statistical significance in tuberculous pleurisy. The values of CD8 were similar in the pleural fluid and in blood. Levels of CD16 were non-significantly higher in pleural fluid in all groups.

**CONCLUSION:**

This study confirms the hypothesis that pleural cavity is compartment with immunological reactivity and results could be used in differential diagnosis together with other examinations.

## Introduction

Lymphocytes are the primary effectors of cellular and humoral immunocompetence in humans. Lymphocytic pleural effusions are characterized by divergent cellular responses depending on the etiology of disease [1]. The accumulation of fluid in the pleural space indicates the presence of systemic or local disease. Pleural exudates involve the migration of immune cells to the pleural cavity [2]. Lymphocytes dominance occurs in the most chronic pleural effusions [3, 4]. The proportion of T and B lymphocytes in pleural fluids relative to that in peripheral blood may point to the presence of local immunological phenomena in various pulmonary and pleural disorders. Tuberculosis and malignant disease are among most frequent causes of pleural effusions. In both causes, the pleural fluid is generally lymphocytic, with predominance of T lymphocytes, particularly CD4+ positive T cells [2, 5, 6]. Malignant effusions are a relatively easily accessible source of tumor-associated T cells and this represent a suitable model for the study of interactions between tumor cells and the host immune system [7].

Considering the compartmentalization of the pleural space, the association between the local and systemic cellular responses should be analyzed.

## Material and Methods

We have investigated the distribution of T and B lymphocytes, T cell with helper/inducer CD4+ or suppresser/cytotoxic CD8+ phenotypes and the subset of cells with natural killer NK activity. We have used the analysis of T cell subsets by monoclonal antibodies defined markers. We studied 48 patients from Clinic of Pulmonology and Allergology with pleural effusions, divided in three groups. First group had 18 patients with tuberculous pleural effusions, Second group had 20 patients with malignant pleural effusions (mesothelioma, lung carcinoma or metastatic pleural effusions) and third group had 10 patients with transudates secondary to cardiac failure. We have also examined a group of 30 healthy controls.

In our study we evaluated:

1) the frequency of lymphocyte predominance in different malignant and non-malignant pleural effusions;
2) lymphocyte phenotype and the ratio between helper (CD4+) and cytotoxic/suppressor (CD8+) lymphocytes in malignant and non-malignant effusions.

Results were statistically elaborated according to the Student t-test and Analysis of Variance (ANOVA).

## Results

According to our results there were a significant decrease of the levels of CD3, CD4, CD16 and CD22 positive cells in peripheral blood of patients with tuberculous pleural effusions versus healthy controls (Table 1). It is due to suppresser activity of lymphocytes in peripheral blood, but also to the immunological reactivity of the pleural effusions.

**Table 1:** Values of CD markers in blood of patients with tuberculous effusion versus healthy controls

	CD3%	CD4%	CD8%	CD16%	CD22%	CD4/CD8
Controls (N=30)	65	38	23	15	15	1.65

Tuberculous (N=18)	59.33	31.66	22	12.67	11.78	1.44

t-test	2.31	2.91	0.56	2.09	2.06	

Significance	S	S	NS	S	S	

**Figure 1:**
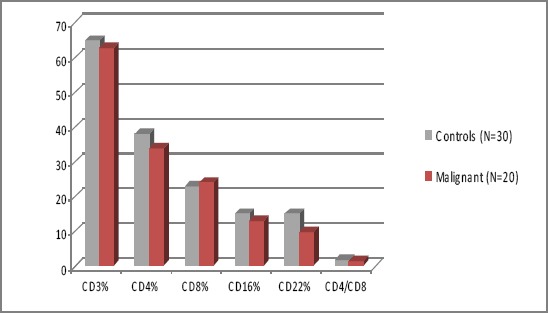
*Values of CD markers in the peripheral blood of patients with malignant pleural effusions versus values of CD markers in peripheral blood in healthy controls*.

Evaluation of the values of CD markers in peripheral blood with malignant effusions versus healthy controls, demonstrate significant decrease in patients with malignant effusions only for CD22 cells. Changes of the values for CD3, CD4, CD8 and CD16 T cells were not significant (Fig. 1)

Values of CD markers in peripheral blood with transudates versus healthy controls demonstrate significant decrease in patients with transudates only for CD22 cells. Changes of the values for CD3, CD4, CD8 and CD16 T cells were not significant (Table 2).

**Table 2:** Values of CD markers in blood of patients with transudates versus healthy controls

	CD3%	CD4%	CD8%	CD16%	CD22%	CD4/CD8%
Controls (N=30)	65	38	23	15	15	1.65

Transudates (N=10)	59.6	33.2	22.6	13.4	9.2	1.6

T-test	1.63	0.62	0.17	1.36	3.21	

Significance	NS	NS	NS	NS	S	

**Figure 2:**
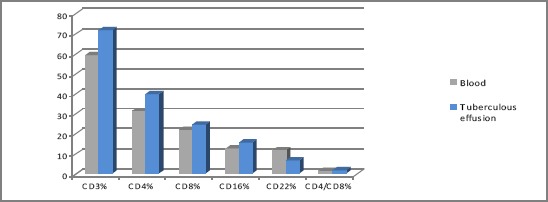
*Values of CD markers in blood and pleural effusions in patients with tuberculous pleurisy*.

In our study, the analysis of the values of CD markers in patients with tuberculous effusions, demonstrate significant increase of the percentage of CD3 and CD4 in the pleural fluid versus blood. We also noticed significant decrease of CD22 cells in pleural tuberculous effusion (p<0.05) (Fig. 2).

In patients with malignant effusions, we noticed increase of the values of CD3, CD4, CD8 and CD16 cells in the pleural malignant fluid versus blood, but these changes were not significant, (p<0.05) (Table 3).

**Table 3:** Comparison of values of CD markers in blood and pleural effusions in patients with malignant effusion

	CD3%	CD4%	CD8%	CD16%	CD22%	CD4/CD8%
Blood	63	33.8	24	13	9.7	1.41

Malignant effusion	67.68	38.63	25.37	15.67	7.67	1.52

T-test	1.18	1.62	0.66	1.42	1.26	

Significance	NS	NS	NS	NS	NS	

**Figure 3:**
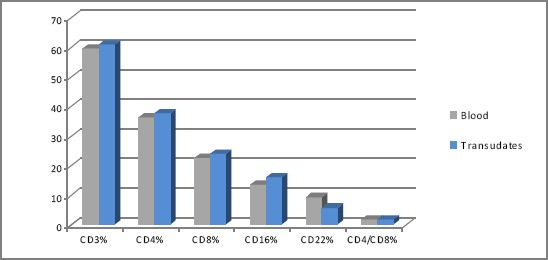
*Values of CD markers in blood and pleural effusions in patients with transudates*.

According to our results, in patient with transudates, the values of CD3, CD4, CD8 and CD16 cells were not significantly changed, (p<0.05) (Fig. 3).

## Discussion

This study evaluated the concept of compartments and it also investigated the significance of immunological reactivity of pleura. In our study, according to our results, there was a significant decrease of the levels of CD3, CD4, CD16 and CD22 positive lymphocyte cells in peripheral blood of patients with tuberculous pleural effusion versus healthy controls. Shiratusci in his study demonstrate the evaluation of the values of the lymphocyte subsets and there are noticed the decrease of values of CD3 and CD4 cells in patients with tuberculosis versus healthy subjects [8]. Compared with controls, the cases showed total lymphocytopenia in peripheral blood and also major depletion of the peripheral T lymphocytes [9].

According to our results, in patients with malignant pleural effusion we noticed decreased values for CD3, CD4 and CD16 positive T cells versus healthy subjects and increases for CD8 positive T cells, but there were not significant. We noticed significant decrease only for CD22 lymphocytes. In literature it was observed lower percentage of CD4+ lymphocytes subsets and higher percentage of CD8+ lymphocytes subsets in malignant when compared to non-malignant fluids [10]. Values of CD markers in peripheral blood with transudates versus healthy controls demonstrate significant decrease in patients with transudates only for CD22 cells. Changes of the percentage for CD3, CD4, CD8 and CD16 positive T cells were not significant due to low inflammatory reactivity in the pleural space of patients with cardiac failure.

In our study, the analysis of the values of CD markers in patients with tuberculous effusions, demonstrate significant increase of the values of CD3 and CD4 positive cells in the pleural fluid versus blood. We also noticed significant decrease of the percentage of CD22 cells in pleural tuberculous effusion versus blood. In literature, it is demonstrated that pleural involvement is associated with migration of immune cells to the pleural cavity [2] and it is noticed the predominance of T helper cells into pleural space [11-13]. The lymphocytic subpopulation study confirms the concept of compartmentalization in tuberculous pleuritis, as shown by the greater number of activated T lymphocytes present in pleural fluid in comparison with peripheral blood in patients with tuberculous pleural effusions [14]. and predominance of helper cells (CD4+) in pleural fluid [12, 14, 15]. According our study the CD4/CD8 ratio was greater in pleural fluid then in peripheral blood as it is known in literature [14].

In patients with malignant effusions, we noticed increase of the values of CD3, CD4, CD8 and CD16 positive T cells in the pleural malignant fluid versus blood, but these changes were not significant due to different etiologies of malignancy. Some authors demonstrate increased CD4+ T lymphocyte subset in malignant pleural effusion [16, 17]. In the literature there are similar findings with explanation that it is due to suppresser activity of lymphocytes in peripheral blood, but also to the immunological reactivity of the pleural effusions [18]. In malignant effusions, the inflammatory processes and the immune responses induce the recruitment of cells into the pleural space [19]. The levels of CD16 (NK cells) were non-significantly higher in pleural fluid in all three groups and it show that they have not a relevant role in immunological reactivity of the pleura and diagnosis of pleurisy. Some authors found that despite a higher percentage of circulating NK cells in patients with pleural malignancies than in healthy subjects, there was a defect in recruiting NK cells in malignant pleural effusions [11, 20].

The patients with transudates secondary to cardiac failure have very small difference of the values versus other groups that is according to low immunological and inflammatory reactivity in the pleural space of these patients.

In conclusion, the values of CD3 and CD4 positive T cells were significantly higher in the pleural fluid of patients with tuberculous pleural effusions. The results suggest that the responding T lymphocytes have been portioned from the peripheral blood to the site of inflammation. The lymphocytes chemoattractans are present in the pleural fluid and these factors enhance the accumulation of T cells to the pleural cavity (especially in patients with tuberculous pleuricity). The patients with transudates secondary to cardiac failure have very small difference between the percentages in all three groups. We can say that this study confirms the hypotheses that the pleural cavity is compartment with its own immunological reactivity. The results of this study could be used in differential diagnosis only together with other clinical and biochemical examinations.

## References

[1] Dalbeth N, Lee YCG (2005). Lymphocytes in pleural disease. Current Opinion in Pulmonary Medicine.

[2] Aguiar LMZ, de Antonangelo L, Vargas FS, Zerbini MCN, Sales MM, Uip DE, Saldiva PHN (2008). Malignant and tuberculous pleural effusions:immunophenotypic cellular characterization. Clinics.

[3] Kochman S, Bernard J, Lavaud F, Cazabat A, Dubois Montreynaud JM (1984). T lymphocyte subsets in pleural fluids:discrimination according to traditional and monoclonal antibody-defined markers. Eur J Resp Dis.

[4] Lucivero G, Pierucci G, Bonamo L (1988). Lymphocyte subsets in peripheral blood and pleural fluid. Eur Resp J.

[5] Valdés L, Pose A, San José E, Martínez Vázquez JM (2003). Tuberculous pleural effusions. Eur J Intern Med.

[6] Masakazu O, Yoshinori H, Toru H, Naozumi H, Kazuyoshi I, Kaoru Sh, Tsutomu K (2005). T-Helper Type 1/T-Helper Type 2 Balance in Malignant Pleural Effusions Compared to Tuberculous Pleural Effusions. CHEST.

[7] Atanackovic Dj, Block A, De Weerth A, Faltz C, Hossfeld DK, Hegewisch-Becker S (2004). Characterization of Effusion-Infiltrating T Cells:Benign versus Malignant Effusions. Clinical Cancer Research.

[8] Shiratsuchi H, Tsuyguchi J (1984). Analysis of T cell subsets by monoclonal antibodies in patients with tuberculosis after in vitro stimulation with purified protein derivate of tuberculin. Clin Exp Immunol.

[9] Ghosha AG, Mukherjee K, Saha AK, Chowdhury TK (1996). Compartmentalisation of lymphocytes in tuberculous pleural effusion:a preliminary observation. Ind J Tub.

[10] Klimiuk J, Domagała-Kulawik J, Krenke R, Chazan R (2004). Lymphocyte and lymphocyte subsets in pleural fluid - comparison of malignant and non-malignant disorders. Pol Arch Med Wewn.

[11] Barnes PF, Mistry SD, Cooper CL, Pirmez C, Rea TH, Modlin RL (1989). Compartmentalization of a CD4+T lymphocyte subpopulation in tuberculous pleuritis. J Immunol.

[12] Trajman A, Pai M, Dheda K, van Zyl Smit R, Zwerling AA, Joshi R, Kalantri S, Daley P, Menzies D (2008). Novel tests for diagnosing tuberculous pleural effusion:what works and what does not?. Eur Respir J.

[13] Tong ZH, Shi HZ (2013). Subpopulations of helper T lymphocytes in tuberculous pleurisy. Tuberculosis.

[14] San José ME, Valdés L, Saavedra MJ, De Vega JM, Alvarez D, Vi-uela J, Penela P, Valle JM, Seoane R (1999). Lymphocyte populations in tuberculous pleural effusions. Ann Clin Biochem.

[15] Qin XJ, Shi HZ, Liang QL, Huang LY, Yang HB (2008). CD4+CD25+regulatory T lymphocytes in tuberculous pleural effusion. Chin Med J (Engl).

[16] Robinson E, Segal R, Vesely Z (1986). Lymphocyte subpopulation in peripheral blood and malignant effusions of cancer patients. Eur J Cancer Clin Oncol.

[17] Chen YQ, Shi HZ, Qin XJ, Mo WN, Liang XD, Huang ZX, Yang HB, Wu C (2005). CD4+CD25+regulatory T lymphocytes in malignant pleural effusion. Am J Respir Crit Care Med.

[18] Michael RS, David WE (1986). T lymphocytes and pleural tuberculosis. Chest.

[19] Scherpereel A, Grigoriu BD, Noppen M, Gey T, Chahine B, Baldacci S, Trauet J, Copin MC, Dessaint JP, Porte H, Labalette M (2013). Defect in recruiting effector memory CD8+T-cells in malignant pleural effusions compared to normal pleural fluid. BMC Cancer.

[20] Onishi H, Morisaki T, Kuga H, Katano M, Doi F, Uchiyama A, Sugitani A, Wada J, Chijiiwa K, Tanaka M (2002). A large quantity of CD3-/CD19-/CD16- lymphocytes in malignant pleural effusion from a patient with recurrent cholangio cell carcinoma. Immunol Invest.

